# Comparing the association of two metabolic syndrome definitions, NCEP ATP III and IDF, with the risk of developing atherosclerotic cardiovascular disease: An analytical cross‐sectional study

**DOI:** 10.1002/edm2.468

**Published:** 2024-01-17

**Authors:** Gholamreza Yousefzadeh, Amin Sayyadi, Hamid Najafipour, Vida Sabaghnejad, Sara Pezeshki

**Affiliations:** ^1^ Endocrinology and Metabolism Research Center Institute of Basic and Clinical Physiology Sciences, Kerman University of Medical Sciences Kerman Iran; ^2^ Department of Internal Medicine Kerman University of Medical Sciences Kerman Iran; ^3^ Student Research Committee, School of Medicine Kerman University of Medical Sciences Kerman Iran; ^4^ Cardiovascular and Respiratory Physiology, Cardiovascular Research Center Institute of Basic and Clinical Physiology Sciences, Kerman University of Medical Sciences Kerman Iran; ^5^ Physiology Research Center Institute of Neuropharmacology, Kerman University of Medical Sciences Kerman Iran

**Keywords:** atherosclerotic cardiovascular diseases, IDF, metabolic syndrome, NCEP ATP III

## Abstract

**Introduction:**

Atherosclerotic cardiovascular diseases (ASCVD) are significant sources of mortality and morbidity with substantial economic implications and preventive measures play key roles in this regard. Metabolic syndrome (MetS) is a common condition, and its association with ASCVD and mortality has made it clinically important. However, controversies persist regarding the best definition for MetS. Here in, we investigated the ability of the International Diabetes Federation (IDF) and the National Cholesterol Education Program Adult Treatment Panel III (NCEP ATP III) in the prediction of ASCVD incidence.

**Methods:**

We conducted an investigation on individuals diagnosed with MetS as part of the “Kerman Coronary Artery Diseases Risk Factor Study” (KERCADRS). This study was a cohort study conducted on a population aged 15–75 years residing in Kerman, Iran to assess the risk of ASCVD. We employed ACC/AHA ASCVD Risk Estimator for predicting ASCVD occurrence in the future and then compared the results with different definitions of MetS including IDF and NCEP ATP III.

**Results:**

Patients with MetS consistent with NCEP ATP III had higher ASCVD risk scores than those with IDF (10.63 ± 10.989 vs. 9.50 ± 9.357). NCEP ATP III had better overall performance in terms of specificity, accuracy, sensitivity and positive and negative predictive values especially in higher ASCVD risk score categories. The agreement between IDF and NCEP ATP III was none to slight (Cohen's Kappa <0.2) except for IDF in the group of ASCVD >30%, which revealed no agreement (Cohen's Kappa = 0).

**Conclusion:**

NCEP ATP III has better overall performance compared to IDF. The ability of NCEP ATP III increases as the ASCVD risk score goes higher. IDF may be useful in primary screening and patients with lower ASCVD risk scores.

## Graphical abstract

We conducted an analytical cross‐sectional study to assess the ability of NCEP ATP III and IDF in predicting ASCVD in the future. We realised that NCEP ATP III has better overall ability in this regard.

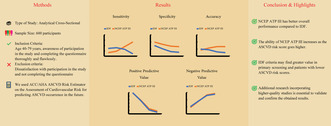

## 1 INTRODUCTION

Atherosclerotic cardiovascular diseases (ASCVD) are the number one cause of mortality and morbidity globally.^1^ In 2020, almost 19 million people died from ASCVD.^2^ The economic burden of ASCVD is substantial, and it's still increasing. Prevention is a crucial factor and can be achieved through practicing healthy habits: physical activity, healthy diets, non‐smoking, reduced alcohol consumption and reduced stress.^3^

Obesity‐associated cardiovascular risk factors including abdominal obesity, hypertriglyceridemia, decreased high‐density lipoprotein (HDL), high blood pressure, and/or impaired glucose tolerance, are the elements of metabolic syndrome (MetS). The prevalence ranges from 20%–25% in adults and 0%–19.2% in children; up to 80% of diabetic patients can have MetS.^4^ The trend is also rising: the prevalence of MetS in the United States (US) adults has increased from 25.3% in 1988–1994 to 34.2% in 2007–2012.^5^ High prevalence, rising trend and association with ASCVD and mortality have made MetS a clinically important issue.^6^

Multiple definitions are available for MetS including World Health Organization (WHO), European Group of Insulin Resistance (EGIR), International Diabetes Federation (IDF) and National Cholesterol Education Program Adult Treatment Panel III (NCEP ATP III); these definitions have shown different abilities.^7^ The first definition was proposed by WHO in 1998, which believed that insulin resistance and its surrogates, impaired glucose tolerance (IGT), and type 2 diabetes mellitus, are the essential components of MetS; other components consisted of raised blood pressure, hypertriglyceridemia or low HDL, obesity and microalbuminuria. In IDF's definition of MetS (2005), central obesity is the prerequisite of the MetS diagnosis. The IDF has a particular emphasis on waist measurement as a simple screening tool. The NCEP ATP III (2001) had a different approach; neither insulin resistance nor central obesity were the essential part of the MetS definition; instead, the NCEP ATP III definition approached MetS as a collection of metabolic factors and remained more silent regarding the exact pathophysiology.^8^

Some studies^9^, ^10^, ^11^, ^12^ have assessed different definitions, but controversies still remain. Although the main components of MetS in different criteria are the same, the existence of some critical differences and having different thresholds have caused disparities between the results of different definitions.

Assessing baseline ASCVD risk using ASCVD risk assessment tools is the beginning of the foundation of preventive cardiology. By using the ASCVD risk assessment tools, we can determine the risk of developing ASCVD in individuals and try to prevent ASCVD in them. Usually, people with low risk are encouraged to do lifestyle modifications, and those with higher risks are recommended to have both lifestyle modifications and pharmacologic therapy.^13^ ASCVD risk score^14^ is one of these tools provided by the American College of Cardiology (ACC)/American Heart Association (AHA). The score is the result of evaluating the risk of having ASCVD in 10 years.

Due to the discrepancy in the literature between different definitions of MetS and different populations, we decided to conduct a cross‐sectional study to find out whether IDF or NCEP ATP III definition can better predict the risk of ASCVD in the Iranian population.

## 2 METHODS

### 2.1 Participants and procedures

We conducted an analytical cross‐sectional study to compare the association of IDF with NCEP ATP III with ASCVD incidence. The population consisted of all the individuals with the diagnosis of MetS in the “Kerman Coronary Artery Diseases Risk Factor Study” (KERCADRS),^15^ which was a cohort study performed on the population aged 15–75 years living in Kerman, Iran (the largest city in south‐eastern Iran with a population of about 780,000 people) by Physiology Research Center of Kerman University of Medical Sciences (KPRC) in two phases: September 2009–December 2011 and October 2014–September 2018. Inclusion criteria in the present analysis included age 40–79 years, awareness of participation in the study and completing the questionnaire thoroughly and flawlessly. Dissatisfaction with participation in the study and not completing the questionnaire were the exclusion criteria. By using simple random sampling method, two groups with MetS based on IDF and NCEP ATP III definitions were selected and matched in terms of age, gender and cigarette smoking (Table 1).

**TABLE 1:** Defining ATP III and IDF as the definitions of metabolic syndrome.

	NCE ATP III	IDF
Criteria	At least any three of the five criteria below	Number 1 + at least any two of the criteria below
1	Waist circumference: >102 cm (M), >88 cm (F)	Central obesity (waist circumference): ≥94 cm (M), ≥80 cm (F)
2	Fasting glucose ≥110 mg/dL or Rx	Fasting glucose ≥100 mg/dL or Rx
3	TG ≥150 mg/dL or Rx	TG ≥150 mg/dL or Rx
4	HDL: <40 mg/dL (M), <50 mg/dL (F) or Rx	HDL: <40 mg/dL (M), <50 mg/dL (F) or Rx
5	≥130 mm Hg systolic or ≥ 85 mm Hg diastolic or Rx	≥130 mm Hg systolic or ≥ 85 mm Hg diastolic or Rx

Abbreviations: BMI, Body Mass Index; Chol cholesterol; F, Female; HDL, High‐density Lipoprotein; IDF, International Diabetes Federation; IGT, Impaired Glucose Tolerance; IR, Insulin Resistance; M, Male; NCEP ATP III, National Cholesterol Education Program Adult Treatment Panel III; Rx, on treatment; TG, Triglycerides.

### 2.2 Measures

We used ACC/AHA ASCVD Risk Estimator^16^ on the Assessment of Cardiovascular Risk for predicting ASCVD occurrence in the future. It uses variables including age, sex, race, systolic and diastolic blood pressure, total cholesterol, low‐density lipoproteins (LDL), HDL, history of diabetes and smoking, being on treatment for hypertension, and being on statin or aspirin. The resulting score will show whether the person's 10‐year risk for ASCVD is low (<5%), borderline (5%–7.4%), intermediate (7.5%–19.9%) or high (≥20%).

### 2.3 Statistical analysis

SPSS version 18 was our statistical analysis software. For descriptive statistics frequency, relative frequency, and mean were used. To perform inferential analysis, we used the chai square test for categorical variables and the independent *t*‐test for continuous variables. Kappa agreement coefficient was carried out to assess concordance between MetS definitions.

### 2.4 Ethical considerations

This project was reviewed and approved by the ethics committee of the Kerman University of Medical Sciences with the licence number IR.KMU.AH.REC.1397.170. Also, informed consent was obtained from all the participants.

## 3 RESULTS

This study included 600 people (243 men, 40.5%) with mean ± SD age of 56.85 ± 9.5 years. There were more people with MetS consistent with IDF (267, 44.5%) than with NCEP ATP III (228, 38%). Hypertension was the most common condition among the participants (249, 41.5%) and diabetes mellitus was the least common (132, 22%). Sixty‐nine patients (11.5%) were cigarette smokers (Table 2).

**TABLE 2:** Prevalence of underlying condition among participants.

Feature	(*N*, %)
Dyslipidemia	249 (41.5)
Rx for dyslipidemia	159 (67.9)
Hypertension	318 (53)
Rx for Hypertension	219 (68.9)
Diabetes Mellitus	132 (22)
Rx (non‐insulin treatment) for diabetes mellitus	93 (70.5)
Rx (insulin treatment) for diabetes mellitus	21 (15.9)
Cigarette smoker	69 (11.5)

Abbreviations: N, number; Rx, on treatment.

The 10‐year ASCVD risk score was in intermediate range (8.43 ± 9.4741%), ranging from 0.2% to 49.9% (Table 3).

**TABLE 3:** Mean ± SD of measured variables of the participants.

Variable	Mean ± SD	Min–Max
Waist circumference (cm)	89.88 ± 11.197	62–122
SBP/DBP (mmHg)	126.55 ± 18.939/82.79 ± 11.894	85/60–190/120
FBS (mg/dl)	102.7 ± 35.614	70–325
TG (mg/dl)	167.34 ± 69.918	50–400
HDL (mg/dl)	60.44 ± 11.846	36–95
LDL (mg/dl)	117.28 ± 56.237	23/8–212
Chol (mg/dl)	207.99 ± 39.816	112–326
10‐year ASCVD risk score (%)	8.43 ± 9.4741	0.2–49.9

Abbreviations: ASCVD, Atherosclerotic Cardiovascular Disease; Chol, Cholesterol; FBS, Fasting Blood Sugar; HDL, High‐density Lipoproteins; LDL, Low‐density Lipoproteins; SBP/DBP, Systolic Blood Pressure/Diastolic Blood Pressure; TG, Triglyceride.

We used the AHA calculator for assessing cardiovascular risk; using the *t*‐test we realised that the ASCVD risk score was significantly higher in both groups with MetS (p−value<.001), but the difference between those with MetS and those without it was higher in the NCEP ATP III group. Patients with MetS consistent with NCEP ATP III had higher ASCVD risk scores than those with IDF (10.63 ± 10.989 vs. 9.50 ± 9.357; Table 4).

**TABLE 4:** ASCVD risk score in subjects with and without MetS in the two definitions.

	MetS (Mean ± SD)		p−value
Definition	No	Yes	
IDF	7.58 ± 9.494	9.50 ± 9.357	<.001
NCEP ATP III	7.09 ± 8.139	10.63 ± 10.989	<.001

Abbreviations: ASCVD, Atherosclerotic Cardiovascular Disease; IDF, International Diabetes Federation; MetS, metabolic syndrome; NCEP ATP III, National Cholesterol Education Program Adult Treatment Panel III.

**FIGURE 1:**
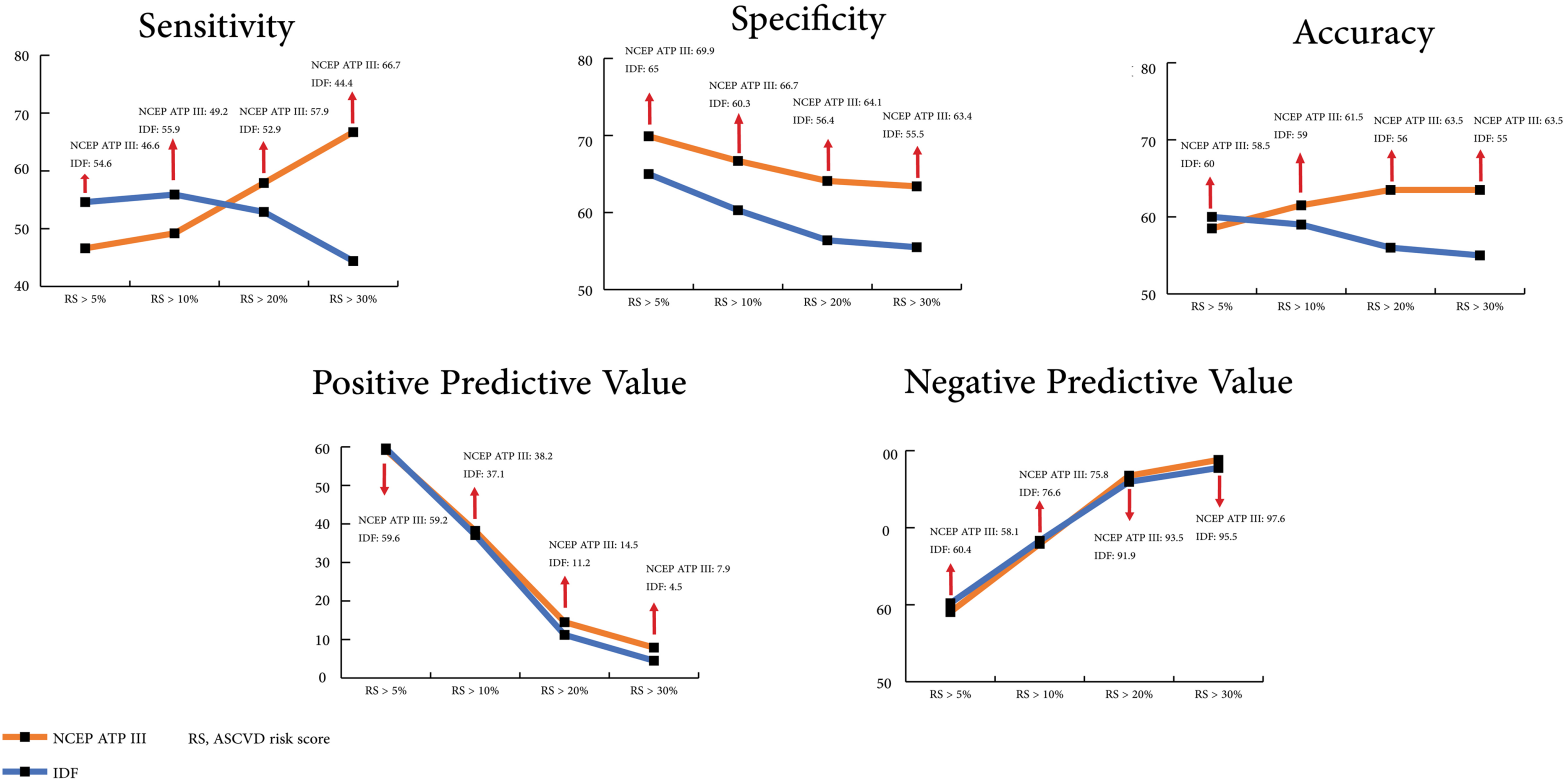
Diagnostic ability of ATP III and IDF in different categories of ASCVD risk scores.

The NCEP ATP III demonstrated superior specificity across all ASCVD risk score groups compared to the IDF criteria. While initially, sensitivity, accuracy, and positive and negative predictive values favoured IDF, as the ASCVD risk scores increased, NCEP ATP III exhibited higher values for these parameters (Figure 1).

The agreement between IDF and NCEP ATP III was none to slight for all ASCVD risk scores (Cohen's Kappa <0.2) except for IDF in the group of ASCVD >30%, which revealed no agreement (Cohen's Kappa = 0).

## 4 DISCUSSION

We performed a cross‐sectional study on 600 patients selected from the previously published cohort: KERCADRS. The ASCVD risk score was significantly higher in patients with MetS in both groups, but the difference was higher for the NCEP ATP III. Although both IDF and NCEP ATP III are weakly correlated with the ASCVD risk score, NCEP ATP III is slightly better at predicting ASCVDs, especially with the fact that, as the ASCVD risk score goes higher, the ability of IDF decreases.

Zibaeenezhad et al.^17^ compared the ability of four definitions of MetS including World Health Organisation (WHO), NCEP ATP III, AHA and IDF in predicting 10‐year ASCVD risk in 7225 patients; they used both ASCVD risk score and Framingham risk score (FRS) for predicting the risk of developing ASCVD in the future; the number of patients diagnosed with WHO criteria was the lowest (*N* = 1676); the difference between patients with and without MetS was statistically significant in AHA, WHO and NCEP ATP III, but the difference was not significant in IDF; they concluded that IDF is not an appropriate criterion for discrimination of patients with and without MetS.

Tsai et al.^18^ conducted a study to investigate the ability of the adjusted ATP III by the American Heart Association and the National Heart, Lung, and Blood Institute (ATP III/AHA/NHLBI) and IDF in predicting ASCVD development. They used brachial‐ankle pulse wave velocity as their ASCVD risk assessment tool; their results showed that the performance of the ATP III/AHA/NHLBI is superior to the IDF.

Moreno et al.^19^ performed a study to evaluate the clinical performance of WHO, NCEP‐ATP III, IDF, AHA/NHLBI, Joint Interim Statement (JIS), and Latin American Diabetes Association (ALAD). They used ACC/AHA ASCVD Risk Calculator as their assessment tool; they realized that the number of patients with MetS according to different definitions varied significantly; JIS, IDF and ALAD criteria diagnosed more patients than others, but WHO and NCEP ATP III diagnosed patients with higher risk.

The prevalence of patients with MetS was higher in IDF than in NCEP ATP III (267, 44.5% vs. 228, 38%). The prevalence of MetS is varied between different studies. In France, Vernay et al.^20^ mentioned that the prevalence of MetS was 14.1%, 16.6%, 20.3% and 21.1% according to NCEP ATP III, AHA/NHLBI, IDF and JIS definitions, respectively. Gündogan et al.^21^ found that the prevalence of MetS in Turkey was 34.6% and 28.8% according to IDF and NCEP ATP III, respectively. Salas et al.^22^ in Mexico used the census definition (IDF/NHLBI/AHA/WHF/IAS/IASO)^23^; they found the prevalence of MetS to be 54.8%. In the United States, Hirode and Wong^24^ used NCEP ATP III and declared the prevalence of MetS to be 34.8%. In Iran, Zibaeenezhad et al.^17^ performed a cross‐sectional analysis of a prospective cohort and found that the prevalence of MetS was 49.6%, 23.2%, 45.5% and 68.4% according to AHA, WHO, NCEP ATP III and IDF. Differences between different populations and using different definitions of MetS are the potential roots of these differences.

Hypertension was the most common underlying condition (53% of the population); following that, dyslipidemia, diabetes mellitus and being a cigarette smoker were present in 41.5%, 22% and 11.5%. Marbou and Kuete^25^ investigated the prevalence of MetS and its components in Cameroon; they found that 32% of 604 patients had MetS, and 58.16%, 6.13%, 3.48% and 11.42% had hypertension, diabetes mellitus and current and former history of smoking; also, decreased HDL (82.78%) and increased LDL (22.85%) were the most and least common forms of dyslipidemia. Biadgo et al.^26^ and Tamiru and Alemseged^27^ worked on the prevalence of MetS and its components in patients with type 2 diabetes mellitus. Biadgo et al. announced the prevalence of MetS at 66.7% in NCEP‐ATP III and 53.5% in IDF. They also reported that ≈55% of their population had elevated blood pressure; dyslipidemia ranges between 64.2% for high cholesterol and 31.5% for low HDL; and 21.3% of the population were current smokers. Tamiru and Alemseged reported the results of a cross‐sectional study on 256 participants; dyslipidemia, Hypertension and being a current smoker were present in 63.5%, 46.5% and 5.5% of the participants. Some studies^28^ have mentioned the prevalence of office elevated blood pressure up to 95.4%; the reason for this high ratio could be that office elevated blood pressure is the mixture of certain cases of hypertension and “white coat” hypertension.

The difference between the ASCVD risk score of patients with and without MetS was significant in both IDF and NCEP ATP III, but the difference was larger in NCEP ATP III; this shows that NCEP ATP III is a better tool to discriminate between individuals with and without MetS. Zibaeenezhad et al. performed the same study and found out that the difference between participants with and without MetS is significant in NCEP ATP III, but the difference was not significant in IDF and declared IDF as an inappropriate tool for diagnosing MetS. The smaller difference in IDF and the larger difference in NCEP ATP III seems to make a difference in the number of patients diagnosed with MetS; as reflected in Moreno et al. study, this can increase the number of patients diagnosed with MetS in IDF and increase the number of higher‐risk patients in NCEP ATP III. Additionally, in our study, NCEP ATP III had higher specificity in all the groups; it also exhibited better performance in terms of sensitivity, accuracy, and positive and negative predictive values.

MetS is closely related to several morbid conditions including ASCVDs and poor kidney function. Topouchian et al.^29^ performed a prospective study on 2224 participants (1664 with MetS, 560 without MetS) aged 40 years and older. They used the cardio‐ankle vascular index (CAVI) and the carotid–femoral pulse wave velocity (CF‐PWV) to evaluate arterial stiffness. They realised that both CF‐PWV and CAVI increase with age, and CAVI had a higher correlation coefficient (comparison of coefficients p−value < .001). With the presence of MetS, age‐adjusted and sex‐adjusted values were significantly higher for CF‐PWV (9.57 ± 0.06 vs. 8.65 ± 0.10, p−value < .001), but such difference was not found for CAVI (8.34 ± 0.03 vs. 8.29 ± 0.04, p−value = .40). Although CF‐PWV was positively associated with all the components of MetS, results were heterogeneous regarding CAVI, which may explain the mentioned difference; CAVI was positively associated with glucose, blood pressure, TG, and HDL, but the correlation was only significant with glucose and blood pressure; in contrast, CAVI had a significantly negative association with waist circumference.

In another study by Scuteri and colleagues^30^ on 6148 participants aged 14–102 years, in any age group, patients with MetS had thicker, stiffer or less distensible, and wider large arteries than participants without MetS. There are differences regarding the effects of different MetS components on arterial changes; any combination of altered glucose tolerance, elevated blood pressure, and elevated TG significantly increased age‐associated arterial alterations. They believed that having MetS accelerates age‐related arterial changes, and this is true even in older patients. Nilsson et al.^31^ characterised arterial ageing by increasing arterial stiffness and measured it with pulse wave velocity (PWV). Participants with healthy vascular ageing (HVA) had significantly lower levels of cardiovascular risk factors including blood pressure, lipids, glucose, obesity, diabetes mellitus, hypertension, and the MetS. They estimated that HVA participants were generally up to 14 years biologically younger than those with higher PWV in terms of vascular health (5.8 ± 0.5 m/s vs. 7.4 ± 1.4; p−value< .0001); they also had lower risk factor levels.

These changes can potentially contribute to greater CVD risk as in a systematic review by Ben‐Shlomo et al.^32^ on 17,635 participants, PWV could reveal itself as an important factor in predicting ASCVDs. PWV was significantly associated with coronary heart disease, stroke, and cardiovascular events in multiple analyses (p−value < .001). Adding PWV to indices could even improve their risk prediction ability in some subgroups (13% for 10‐year CVD risk for intermediate risk).

Additionally, MetS is associated with poorer kidney function. Obesity, as a pivotal component of MetS, predisposes patients to diabetic nephropathy, hypertensive nephrosclerosis, and focal and segmental glomerular sclerosis; it also plays principal roles in the development and progression of chronic kidney disease (CKD). Also, albuminuria is an important risk factor for ASCVDs, and microalbuminuria is an early manifestation of kidney injury and diabetic nephropathy in MetS. Altered levels of adipokines such as leptin and adiponectin, oxidative stress, and inflammation are several obesity‐ and MetS‐induced mechanisms involved in changes in renal physiology and metabolism.^33^

Endothelial dysfunction marks a crucial early stage in the initiation and advancement of atherosclerosis, playing a significant role in the occurrence of microvascular complications. Adipose tissue changes dramatically in obesity and can effectively influence endothelial function through multiple underlying mechanisms including high production of mediators that can impair insulin action in the skeletal muscle like free fatty acids; decreasing adiponectin production, which is associated with improving insulin sensitivity; and producing high amounts of compounds with the ability to affect endothelial function like adipokines that can directly affect vascular homeostasis.^33^ Adipose tissue can also lead to greater ASCVD risk through altered activation of mineralocorticoid receptors (MRs). Extrarenal MRs adjust several mechanisms such as vascular tone, adipogenesis, adipose tissue function, and cardiomyocyte contraction. In mice, the occurrence of MetS components is related to abnormal activation of MRs in the vasculature and in adipose tissue. This aligns with findings in humans, where elevated aldosterone levels are linked to obesity and MetS. These observations imply that disrupted activation of the aldosterone‐MRs system in extrarenal tissues contributes to significant metabolic dysregulation.^34^

## 5 CONCLUSION

Compared to IDF, NCEP ATP III has better overall performance in terms of sensitivity, specificity, accuracy, and positive and negative predictive value, particularly with patients with higher ASCVD risk scores. In contrast, IDF criteria may find greater value in primary screening and patients with lower ASCVD risk scores. Additional research incorporating higher‐quality studies is essential to validate and confirm the obtained results.

## AUTHOR CONTRIBUTIONS

**Gholamreza Yousefzadeh:** Conceptualization (equal); methodology (equal); project administration (equal); writing – review and editing (equal). **Amin Sayyadi:** Software (equal); visualization (equal); writing – original draft (equal); writing – review and editing (equal). **Hamid Najafipour:** Data curation (equal); resources (equal); writing – review and editing (equal). **Vida Sabaghnejad:** Data curation (equal); formal analysis (equal); investigation (equal). **Sara Pezeshki:** Conceptualization (equal); methodology (equal); writing – original draft (equal); writing – review and editing (equal).

## FUNDING INFORMATION

This research did not receive any specific grant from funding agencies in the public, commercial, or not‐for‐profit sectors.

## CONFLICT OF INTEREST STATEMENT

The authors declare that there are no conflicts of interest regarding the publication of this paper.

## ETHICS STATEMENT

Ethics approval and consent to participate Informed consent was received from the patient before starting the work and the study was approved by the ethics committee of Kerman University of Medical Sciences (Code: IR.KMU.AH.REC.1397.170).

## PATIENT CONSENT STATEMENT

Written consent was obtained from the patient regarding publishing this case report in accordance with the journal's patient consent policy.

## Data Availability

We have put the available data in this article, but in case of any questions you can directly contact the corresponding author.
